# Studies on HIV integrase-LEDGF/p75 interaction inhibitory activity of isatine derivative using the alpha screen luminescent proximity assay

**DOI:** 10.1186/1471-2334-12-S1-O5

**Published:** 2012-05-04

**Authors:** Periyasamy Selvam

**Affiliations:** 1 Devaki Amma Memorial College of Pharmacy, Chelembra, Malapuram-673634, Kerala, India

## Background

During the early stage of HIV-1 replication, integrase (IN) plays important roles at several steps, including reverse transcription, viral DNA nuclear import, targeting viral DNA to host chromatin and integration. Previous studies have demonstrated that HIV-1 IN interacts with a cellular lens epithelium-derived growth factor (LEDGF/p75) and that this viral/cellular interaction plays an important role for tethering HIV-1 preintegration complexes (PICs) to transcriptionally active units of host chromatin. Small molecule inhibitors of HIV IN/LEDGF have emerged as promising new class of antiviral agents for the treatment of HIV/AIDS. Present work is to study the small molecule inhibitor of HIV IN/LEDGF.

## Method

Isatine-sulphadimidine derivative (SPIII-5H) selected for these studies. HIV IN/LEDGF interaction inhibition assay performed by ALPHA screen technique, HIV integrase assay investigated by oligonucleotide based assay and molecular modeling studies also carried by using computational methods.

## Results

Lead molecule SPIII-5H inhibits HIV IN/LEDGF interaction (protein-protein interaction) at 10 uM and HIV integrase activity at 6.8 uM. From molecular modeling study indicates that SPIII-5H bind with active site of HIV integrase (DDE), change the conformation and interrupt the binding of HIV integrase with LEDGF.

## Conclusion

SPIII-5H novel class of inhibitors of HIV IN/LEDGF interaction and this lead molecule is suitable for further molecular modifications.

